# Preparation and Dielectric Sensitivity of Polyurethane Composite Fiber Membrane Filled with BaTiO_3_

**DOI:** 10.3390/membranes12040364

**Published:** 2022-03-26

**Authors:** Gang Lu, Changgeng Shuai, Yinsong Liu, Xue Yang, Xiaoyang Hu

**Affiliations:** 1 Institude of Noise and Vibration, Naval University of Engineering, Wuhan 430033, China; chgshuai@163.com (C.S.); liuyinsong1991@163.com (Y.L.); 2 Key Laboratory of Ship Vibration and Noise, Wuhan 430033, China

**Keywords:** polyurethane dielectric elastomer, dielectric properties, dielectric sensitivity, coaxial electrospinning, fiber membrane, BaTiO_3_

## Abstract

Polyurethane dielectric elastomer (PUDE) is considered a potential underwater flexible actuator material due to its excellent designability and environmental tolerance at the molecular level. Currently, the application of the polyurethane elastomer as an actuating material is constrained by such problems as the conflict between various properties such as dielectric properties and modulus and the low level of dielectric sensitivity. This is a common challenge facing polyurethane dielectric research related to the uneven distribution of dielectric fillers in the matrix. Besides, another challenge for the academic circles is the easy agglomeration of micro and nanofillers. Given the above-mentioned background of the application and technical problems, the coaxial electrospinning technology is proposed in this paper. The polyurethane fiber network is constructed with the preferred hydrolysis resistant polyether-Diphenylmethane diisocyanate (MDI) thermoplastic polyurethane elastomer as the matrix material. Dispersed by ultrasound, the micro nano dielectric filler is integrated into polyurethane fiber through the coaxial dual-channel design. Additionally, directional constraint molding is conducted to improve the agglomeration of small-scale particles induced by the loss of mechanical energy in traditional blending. After characterization, the distribution of BaTiO_3_ particles in the fiber bundle is relatively uniform. Compared to the polyurethane dielectric composites prepared by traditional blending (BaTiO_3_-Dielectric Elastomer, BaTiO_3_-DE), the dielectric sensitivity factor of the polyurethane composite fiber membrane (BaTiO_3_-Dielectric Elastomer Membrane, BaTiO_3_-DEM) is enhanced by over 25%; the electrostrictive strain of BaTiO_3_-DEM is boosted by least 10%.

## 1. Introduction

The concept of “electro active polymer” (EAP) was first proposed in 1880 by German scholar Roentgen who discovered that natural rubber film can deform under the action of an electric field [1]. After more than a century of constant development, there has been a considerable range of EAP materials developed and produced in large amounts for the research of new intelligent materials. Generally, EAP materials can be divided into ionic activated EAP and electronic activated EAP following the exact mechanism of deformation.

As a typical variety of electronic EAP materials, dielectric elastomer (DE) demonstrates advantages of significant electric deformation, excellent elasticity and flexibility, high electromechanical conversion efficiency and fast response. For this reason, it has the potential of widespread application in the research of intelligent materials, including bionic mechanical design, structural vibration and noise reduction [2,3,4,5].

Polyurethane dielectric elastomer (PUDE), as a novel variety of intelligent DE materials, presents high polarity, making it prone to orientation polarization under the electric field, high dielectric constant and large electro-strictive strain [6,7,8,9]. Meanwhile, this sort of material is characterized by a fast response (millisecond level), excellent mechanical properties and high environmental tolerance. The flexible actuator made of the material demonstrates structural simplicity, large stress-strain and the high efficiency of electromechanical conversion. Additionally, thermoplastic polyurethane (TPU) can lead to the occurrence of physical cross-linking owing to intermolecular hydrogen bonding. When heated, this material can transform into liquid before returning to an elastomer state because of cooling. This diversifies the method of TPU processing while endowing PUDE with the massive potential for development.

Concerning such intelligent actuating materials as PUDE, the barrier to research lies in how other properties of the material can be balanced with the improvement of its dielectric properties, which is significant to lowering the risk of excessively high actuating voltage [10]. Currently, the most effective solution to the dielectric modification of PUDE is to disperse the dielectric filler into PUDE; however, it remains difficult to prevent the agglomeration of filler in the matrix, as it can often result in an electrical breakdown [11,12]. Simultaneously, there is a sharp increase in the modulus and dielectric loss of the material induced by the presence of filler in large amounts, causing a significant deterioration in the comprehensive properties of the composite dielectric materials [13,14,15]. Hence, balancing the increase in material modulus with the improvement of dielectric properties possessed by PUDE is essential for enhancing the dielectric sensitivity of the material.

In this paper, the introduction of coaxial electrospinning technology is proposed [16]. First, the polyether MDI TPU was adopted as the matrix material intended for the construction of polyurethane dielectric fiber membrane. Then, BaTiO_3_ dielectric filler [17,18] was filled into a polyurethane fiber bundle with the coaxial dual channel under ultrasonic dispersion. Additionally, the directional constraint molding method was employed to alleviate the agglomeration of small-scale particles caused by the loss of mechanical energy in traditional blending. In this way, the limited amount of BaTiO_3_ can perform its dielectric function to the fullest, so as to improve the dielectric sensitivity of polyurethane materials.

## 2. Experiment

### 2.1. The Main Reagents

Table 1 shows the main reagents information used in this paper.

### 2.2. The Main Devices

Table 2 shows the main devices information used in this paper.

### 2.3. The Experimental Process

#### 2.3.1. Preparation of PUDE

(1) Formulation design

Table 3 shows the design formula of PUDE in this paper.

(2) Experimental steps

(1) Prepare materials by complying with the formula in Table 3 and place the mold release agent to the molds with a thickness of 0.5 mm in a 100 °C environment.

(2) Subsequently, pre-mix the PUP and BaTiO_3_ at the temperature of 75 °C, then place them into a shear mixer and regulate at the rotational speed of 5000 r/min for deep mixing (when BaTiO_3_ exists). Label the mixture as material A. Moreover, mix the measured PA, BDO and DABCO evenly at 42 °C, and label the mixture as material B.

(3) Mix A and B. Then, place the mixture in a vacuum oven to remove bubbles. Next, transfer the mixture to the mold and mature at 100 °C for 24 h. Finally, eject the samples out and place them at the ambient temperature for one week for further tests.

#### 2.3.2. Preparation of Polyurethane Fiber Membrane Filled with BaTiO_3_

(1) Technical description and recipe setting

**Figure 1:**
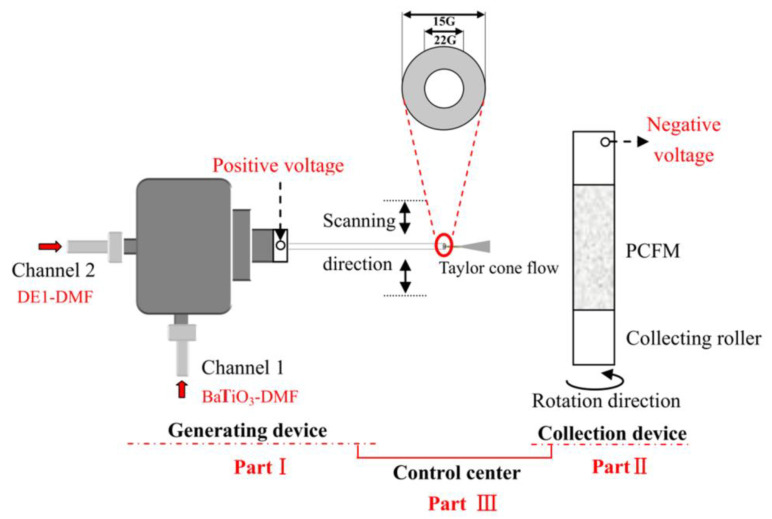
Schematic diagram of coaxial electrospinning technology.

Figure 1 illustrates a schematic diagram of the coaxial spinning device used in this experiment, which consists of a coaxial spinning generator, fiber membrane collection device and electrospinning control center. The mechanism is described as follows. The spinning solution in the two channels exhibited at the left end of the schematic diagram gives rise to Taylor cone flow because of high voltage. Then, the continuous fiber bundle accumulates on the collection roller and ends up with the generation of fiber film. The adjustment can be made to the concentration of the spinning solution, the speed of glue pushing, the size of voltage, the size of pipe diameter, the scanning speed of the spinning head, the distance from pipe orifice to the roller, the direction of rotation for the roller and environmental factors such as temperature and humidity. Furthermore, the micromorphology of the fiber membrane can be adjusted to obtain an ideal polyurethane composite fiber membrane.

DE_1_-DMF is a PUDE solution dissolved by N, N-dimethylformamide; BaTiO_3_-DMF is BaTiO_3_ dispersion by N, N-dimethylformamide suspension; PCFM is the polyurethane composite fiber membrane.

Table 4 presents the component settings of a shaft sleeve and axial spinning solution in the coaxial dual channel. Notably, the thickness and composition of the polyurethane fiber film filled with the BaTiO_3_ prepared in this section (BaTiO_3_-Dielectric Elastomer Membrane, BaTiO_3_-DEM) are consistent with those of BaTiO_3_-DE; the thickness measurement of this part depends on a thickness gauge with an accuracy of 1 μm. In other words, the approach facilitates the comparison in overall performance with BaTiO_3_-DE.

(2) Experimental process

(1) Inject the prepared spinning solution into the shaft axis and shaft sleeve channel, adjust the initial position of the spinning head, fix the tin foil on the collecting roller and then close the safety door when the preparation is complete.

(2) Switch on the control center, and then adjust the distance between the spinning head and the roller, glue pushing speed, high voltage valve, roller speed, scanning starting point and distance successively. Next, the spinning parameters are determined through the shape of the Taylor cone flow of the spinning head.

(3) At the end of spinning, it is confirmed that all parameters of the control center are closed. Then, the safety door is opened. The tinfoil is carefully removed from the roller and transferred to the blast oven. The temperature is set to 30 °C for 12 h. Finally, the fiber film is removed from the tin foil to conduct the characterization test. Among them, BaTiO_3_-DEM_1_ can be prepared using the traditional electrospinning method, which is not detailed in this paper.

## 3. Results and Discussion

### 3.1. Test Method

#### 3.1.1. Characterization Projects

The first step is to examine the micromorphology of the cross-section of the fibrous membrane using the scanning electron microscope to observe the dispersion of BaTiO_3_ in the fiber bundle. The device information is listed in Table 5.

#### 3.1.2. Density and Hardness Test

The density and hardness of BaTiO_3_-DEM of the fully prepared network structure are supposed to be lower than those of BaTiO_3_-DE. Therefore, the density and hardness tests are performed separately.

According to the national standard GB/T4472-2011, the sample mass m is measured with an analytical balance first. Then, the sample volume V is measured with a scale. The density of the sample is obtained using the formula ρ = m/V.

According to the national standard GB/T531.1-2008, the hardness of the sample is examined using the TH-200 hardness tester.

#### 3.1.3. Dielectric Sensitivity Factor Test

The second step is to perform a dielectric sensitivity factor on the polyurethane composite fiber membrane. Considering that the dielectric sensitivity factor β is expressed in Equation (1), the dielectric constant and elastic modulus are tested as follows.

(1) $$\beta =\frac{\varepsilon_{r}}{Y}$$

It can be seen from this equation that for the dielectric material, the greater the dielectric constant and the lower the elastic modulus, the higher the dielectric sensitivity factor. Therefore, it is essential to balance the elastic modulus of materials while improving the dielectric properties of materials.

(1) Dielectric Constant Test

The dielectric properties of the samples are measured using the dielectric constant tester of model 6632-1s from the Teng Skye company. The test frequency ranges from 10 Hz to 500 Hz, the test temperature is room temperature and the sample size is 10 mm.

(2) Elastic Modulus Test

According to the national standard GB/T 528-1998, the sample is cut into multiple 2 mm × 5 mm × 2 mm dumbbell-shaped splines, with the tensile rate as 200 mm/min. The elastic modulus is calculated based on the slope of the initial part (deformation less than 5%) on the stress–strain curve. It is necessary to take the median value of five parallel test values.

#### 3.1.4. Breakdown Voltage and Electrostrictive Strain Test

**Figure 2:**
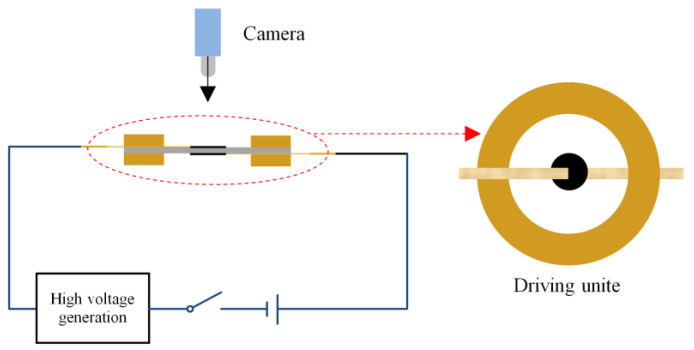
Schematic diagram of electrostrictive strain and electrical breakdown test.

The perfluoropolyether conductive electrodes are uniformly coated on both sides of the dielectric elastomer; then, a simple driving unit is obtained. Next, the variable voltage is applied by the high-voltage generator on the dielectric elastomer coated with the electrode. At this time, the area changes in the dielectric elastomer are recorded using the high-definition digital camera and scale. This phenomenon is called electrostrictive strain. Afterward, the recorded image is extracted and recognized, while the electric deformation of the dielectric elastomer is obtained under different loading voltages. The schematic diagram of electrostrictive strain and the electrical breakdown test are exhibited in Figure 2.

On this basis, the loading voltage is continuously boosted until electric breakdown occurs to the dielectric elastomer. Meanwhile, the voltage is recorded as breakdown voltage.

### 3.2. Result Analysis

(1) Filling state of BaTiO_3_ in fiber bundle

**Figure 3:**
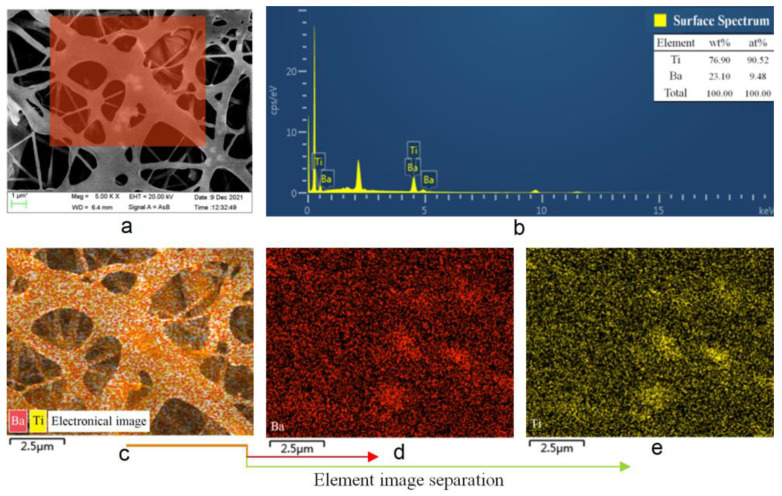
Distribution of BaTiO_3_ in polyurethane fiber membrane prepared by coaxial spinning technology. Among them, (**a**) is the arrangement trend diagram of BaTiO_3_ in polyurethane fiber, (**b**) shows the proportion diagram of Ti and Ba elements, (**c**) is the distribution diagram of Ti and Ba elements, and (**d**,**e**) are the distribution diagrams of Ba and Ti elements respectively.

With BaTiO_3_-DEM_2_ as an example, the diagram of the electron microscope analysis and energy spectrum analysis for the distribution of BaTiO_3_ in polyurethane fiber is presented in Figure 3. The red area in Figure 3a suggests that the white bright spots (BaTiO_3_) present a regional directional arrangement in the fiber bundle. Figure 3b indicates the contents of elements Ti and Ba. Figure 3c shows the corresponding Figure 3a. According to the energy spectrum analysis of the medium red area, the diagram of Ba element distribution and that of Ti element distribution on the right are obtained, respectively, after element separation. It reveals that Ba elements and Ti elements are evenly distributed in the fiber membrane. The above results suggest that BaTiO_3_ is oriented in the fiber bundle, and BaTiO_3_ is basically uniformly distributed in the fiber membrane. According to past research, when BaTiO_3_ is added to the polymer matrix by more than five parts, due to the strong dipole interactions between the polarized BaTiO_3_ spheres, a more pronounced particle aggregation is observed [19], and the properties of composites will be greatly affected. Therefore, it is very meaningful to make the limited amount of BaTiO_3_ uniformly filled in the polyurethane matrix.

(2) Density and hardness

Table 6 shows the density and hardness data of the BaTiO_3_-DE series and BaTiO_3_-DEM series samples, respectively.

According to Table 6, the density and hardness of the BaTiO_3_-DEM series’ materials have declined to varying degrees compared with the BaTiO_3_-DE series’ materials produced through traditional mechanical blending. This is because the BaTiO_3_-DEM series’ materials possess a fiber network structure, and the internal multi void structure plays a role in weakening their density and hardness. This structure contributes to a decrease in the modulus of materials, as listed in Table 7.

(3) Dielectric sensitivity factor

**Figure 4:**
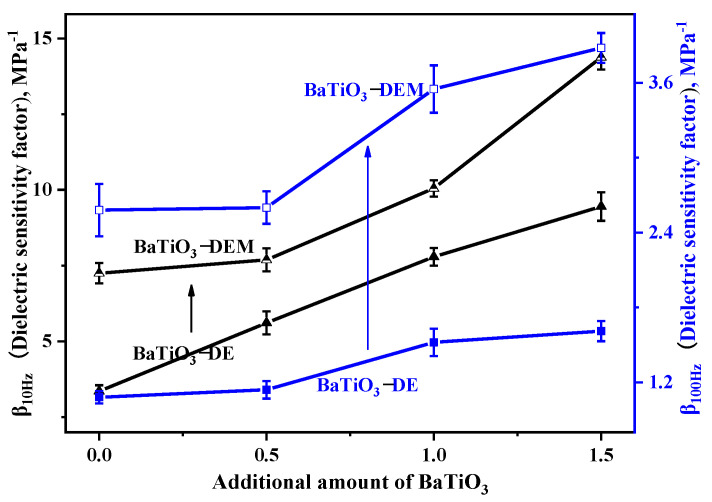
Comparison diagram of dielectric sensitivity factors between two kinds of samples.

The above table shows the dielectric properties, modulus and dielectric sensitivity factor of the BaTiO_3_-DE series and BaTiO_3_-DEM series samples at 10 Hz and 100 Hz. The modulus results are reciprocally substantiated with the above contents. Figure 4 is presented according to the contents in the table to compare the differences in performance between the coaxial spinning fiber film filled with BaTiO_3_ and the dielectric elastomer.

As observed in the above figure, the dielectric sensitivity factor of the BaTiO_3_-DEM series’ materials prepared by coaxial spinning technology is improved by at least 25% compared to the BaTiO_3_-DE material formed by traditional blending. Specifically, the dielectric sensitivity factor is increased by more than 130% when the test frequency and the additional amount of BaTiO_3_ reach 10 Hz and 0.5%. Following the data listed in Table 7, the above results are closely associated with a significant reduction in the modulus of BaTiO_3_-DEM materials produced by coaxial spinning.

(4) Breakdown voltage and electrostrictive strain

**Figure 5:**
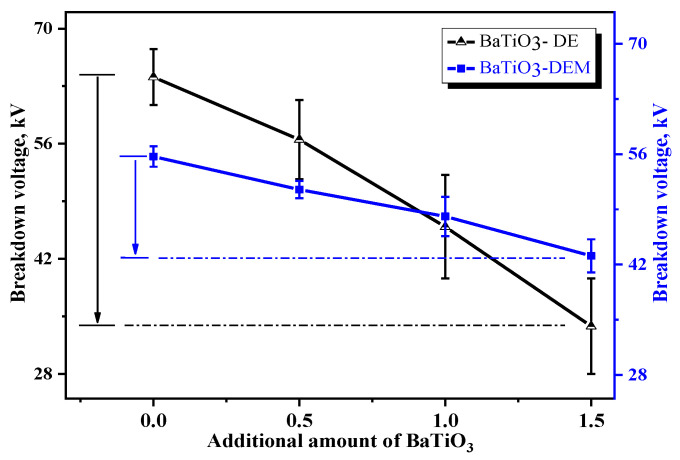
Comparison diagram of breakdown voltage between two kinds of samples.

**Figure 6:**
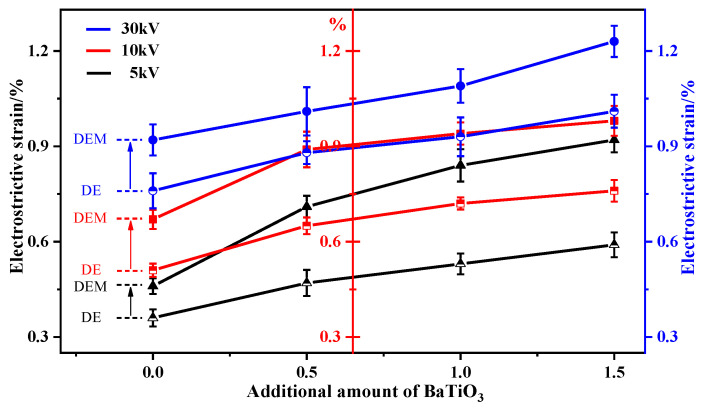
Comparison diagram of electrostrictive strain between two kinds of samples.

Table 8 shows the maximum breakdown voltage and electrostrictive strain of BaTiO_3_-DE series and BaTiO_3_-DEM series samples at 5 kV, 10 kV and 30 kV, respectively. Figure 5 and Figure 6 are presented based on the contents of the table to facilitate the comparison in performance between the coaxial spinning fiber film filled with BaTiO_3_ and the traditional blend dielectric elastomer.

The figure suggests that the maximum breakdown voltage of BaTiO_3_-DE material obtained by the traditional blending method drops significantly with a progressive increase in the amount of the BaTiO_3_ addition. In contrast, the maximum breakdown voltage of the BaTiO_3_-DEM material produced by the coaxial spinning technology reveals barely any reduction. Generally, the breakdown strength is related to the distribution of dielectric fillers in the matrix. The higher the content of micro and nano dielectric fillers, the less difficult the agglomeration of fillers in the matrix, and the easier it is to break down. The results in the figure demonstrate that the application of coaxial spinning technology plays a certain role in improving the distribution of the micro-nano BaTiO_3_ dielectric filler in polyurethane materials.

According to this figure, the electrostrain of BaTiO_3_-DEM material under three voltage loads is more satisfactory compared to the BaTiO_3_-DE material, which is consistent with the magnitude trend exhibited by the dielectric sensitivity factors of these two materials. The increase in the electrostrictive strain of the BaTiO_3_-DEM material is more significant than that of the BaTiO_3_-DE material with the increase in the BaTiO_3_ addition. This verifies that the application of coaxial spinning technology is advantageous over the traditional physical blending method in improving the dielectric properties of comprehensive dielectric materials. The result is directly related to the fact that the dielectric sensitivity factor of BaTiO_3_-DEM is greater than that of BaTiO_3_-DE. In the subsequent research on the electrostrictive properties of a new intelligent material, improving the dielectric sensitivity factor of the composite can be regarded as a research idea which will greatly improve the efficiency of scientific research.

## 4. Conclusions

It is a common and effective method to improve the dielectric sensitivity of the materials by blending the polyurethane dielectric elastomer and micro nano BaTiO_3_ dielectric filler, so as to strengthen its electrostrictive strain for adaptation to the application in intelligent driving. In this paper, the polyurethane fiber membrane filled with BaTiO_3_ is prepared by coaxial spinning technology. The conclusions of this study are drawn as follows.

(1) The dielectric sensitivity of the polyurethane fiber film filled with BaTiO_3_ improves by more than 25% compared with BaTiO_3_-DE obtained by traditional physical blending. Additionally, the breakdown voltage of the former declines by nearly 50% with the increase of BaTiO_3_ content, while that of the latter is merely 22.6%.

(2) Under the voltage loading of 5 kV, 10 kV and 30 kV, the electrostrictive strain of BaTiO_3_-DEM is 10% higher than that of BaTiO_3_-DE at minimum, with some reaching 50%.

## Figures and Tables

**Table 1:** The main reagents.

Reagent	Manufacturer Information	Purity
Polyether-MDI prepolymer (PUP)	Dongguan poly rubber products Co., Ltd., Dongguan, China	CP
Polyether polyol additive (PA)	Dongxu Chemical Industry Manufacturing Co., Ltd., Jiangmen, China	CP
Butanediol (BDO)	Xi’an Tianmao Chemical Co., Ltd., Xi’an, China	AR
Triethylenediamine (DABCO)	Dongxu Chemical Industry Manufacturing Co., Ltd., Jiangmen, China	AR
BaTiO_3_	Huating Chemical Co., Ltd., Shanghai, China	AR

**Table 2:** The main devices.

Device	Manufacturer Information	Remarks
Electric blast drying oven	Yiheng Scientific Instrument Co., Ltd., Shanghai, China	Heating of reagent
Vacuum drying oven	Yiheng Scientific Instrument Co., Ltd., Shanghai, China	Removal of bubbles
Electrospinning apparatus	Tongli micro nano Co., Ltd., Shenzhen, China	Forming of fiber

**Table 3:** Formulation of PUDE.

Formula	A		B		
	PUP (phr)	BaTiO_3_ (ph)	PA (phr)	BDO (phr)	DABCO (phr)
BaTiO_3_-DE_1_	100	0	60	10	0.2
BaTiO_3_-DE_2_	100	0.5	60	10	0.2
BaTiO_3_-DE_3_	100	1.0	60	10	0.2
BaTiO_3_-DE_4_	100	1.5	60	10	0.2

**Table 4:** Setting of a shaft sleeve and axial spinning solution.

Component		DE_1_-DMF (phr)	BaTiO_3_-DMF (phr)
BaTiO_3_-DEM_1_	Traditional electrospinning	17.0–85.0	\
BaTiO_3_-DEM_2_	Shaft sleeve solution	17.0–85.0	\
	Axis center solution	\	0.05–50
BaTiO_3_-DEM_3_	Shaft sleeve solution	17.0–85.0	\
	Axis center solution	\	0.10–50
BaTiO_3_-DEM_4_	Shaft sleeve solution	17.0–85.0	\
	Axis center solution	\	0.15–50

**Table 5:** The device information.

Device	Model	Remarks
Scanning Electron Microscope	Zeiss Merlin	The signal sources were backscattered signal and secondary electron signal; the accelerating voltage was 20 kV.

**Table 6:** Density and hardness between two kinds of samples.

Samples	Density/g.cm^−3^	Hardness/Shore A
BaTiO_3_-DE_1_	1.1203	57.5
BaTiO_3_-DE_2_	1.1542	58.7
BaTiO_3_-DE_3_	1.1685	60.3
BaTiO_3_-DE_4_	1.1917	61.2
BaTiO_3_-DEM_1_	0.8907	24.2
BaTiO_3_-DEM_2_	0.8941	26.5
BaTiO_3_-DEM_3_	0.9057	27.1
BaTiO_3_-DEM_4_	0.9132	27.8

**Table 7:** Dielectric properties and sensitivity factors between two kinds of samples.

Samples	10 Hz		100 Hz		Y/MPa	Dielectric Sensitivity Factor	
	ε_1_”	Tanα_1_	ε_2_”	Tanα_2_		β_10Hz_	β_100Hz_
BaTiO_3_-DE_1_	21.482	0.732	6.930	0.149	6.4 ± 0.2	3.35	1.08
BaTiO_3_-DE_2_	38.704	0.466	7.879	0.109	6.9 ± 0.2	5.61	1.14
BaTiO_3_-DE_3_	58.432	0.578	11.435	0.207	7.5 ± 0.2	7.79	1.52
BaTiO_3_-DE_4_	77.503	0.317	13.208	0.178	8.2 ± 0.2	9.45	1.61
BaTiO_3_-DEM_1_	15.017	0.134	5.347	0.084	2.07 ± 0.08	7.25	2.58
BaTiO_3_-DEM_2_	18.312	0.204	6.163	0.143	2.38 ± 0.08	7.69	2.60
BaTiO_3_-DEM_3_	28.051	0.291	9.906	0.091	2.79 ± 0.08	10.05	3.55
BaTiO_3_-DEM_4_	43.438	0.459	11.765	0.126	3.02 ± 0.08	14.38	3.88

ε: dielectric constant; Tanα: dielectric loss factor.

**Table 8:** Breakdown voltage and electrostrictive strain between two kinds of samples.

Samples	Breakdown Voltage/kV	Electrostrictive Strain/%		
		5 kV	10 kV	30 kV
BaTiO_3_-DE_1_	64.1	0.36	0.51	0.76
BaTiO_3_-DE_2_	56.5	0.47	0.65	0.88
BaTiO_3_-DE_3_	45.9	0.53	0.72	0.93
BaTiO_3_-DE_4_	33.8	0.59	0.76	1.01
BaTiO_3_-DEM_1_	55.7	0.46	0.67	0.92
BaTiO_3_-DEM_2_	51.5	0.71	0.89	1.01
BaTiO_3_-DEM_3_	48.1	0.84	0.94	1.09
BaTiO_3_-DEM_4_	43.1	0.92	0.98	1.23

## Data Availability

The data presented in this study are available on request from the corresponding author.
